# Supplementary material for: Dynamic hinge-motion of PfRIPR revealed by malaria invasion inhibitory antibodies
Source: Nat Commun. 2026 Aug 24;17:10727. doi: 10.1038/s41467-026-77069-z (PMC13658223; doi:10.1038/s41467-026-77069-z)
Supplement: Supplementary file 1 [file 41467_2026_77069_MOESM1_ESM.pdf]

Supplementary Information

Dynamic hinge-motion of PfRIPR revealed by malaria invasion inhibitory antibodies

Brendan Farrell[1,2,†], Andrew J.R. Cooper[3,†], Egle Butkeviciute[1,2], Lawrence T. Wang[3], Emily Egerton-Warburton[1,2], Joshua Tan[3,*] and Matthew K. Higgins[1,2,*]

[1]Department of Biochemistry, University of Oxford, South Parks Road, Oxford OX1 3QU, UK.

[2]Kavli Institute for Nanoscience Discovery, Dorothy Crowfoot Hodgkin Building, University of Oxford, South Parks Rd, Oxford, OX1 3QU

[3]Antibody Biology Unit, Laboratory of Immunogenetics, National Institute of Allergy and Infectious Diseases, National Institutes of Health, Rockville, MD 20852, USA.

[†] contributed equally

[*] for correspondence joshuahoongyu.tan@nih.gov or matthew.higgins@bioch.ox.ac.uk

This PDF includes:

Supplementary Figures 1-8

Supplementary Tables 1-9

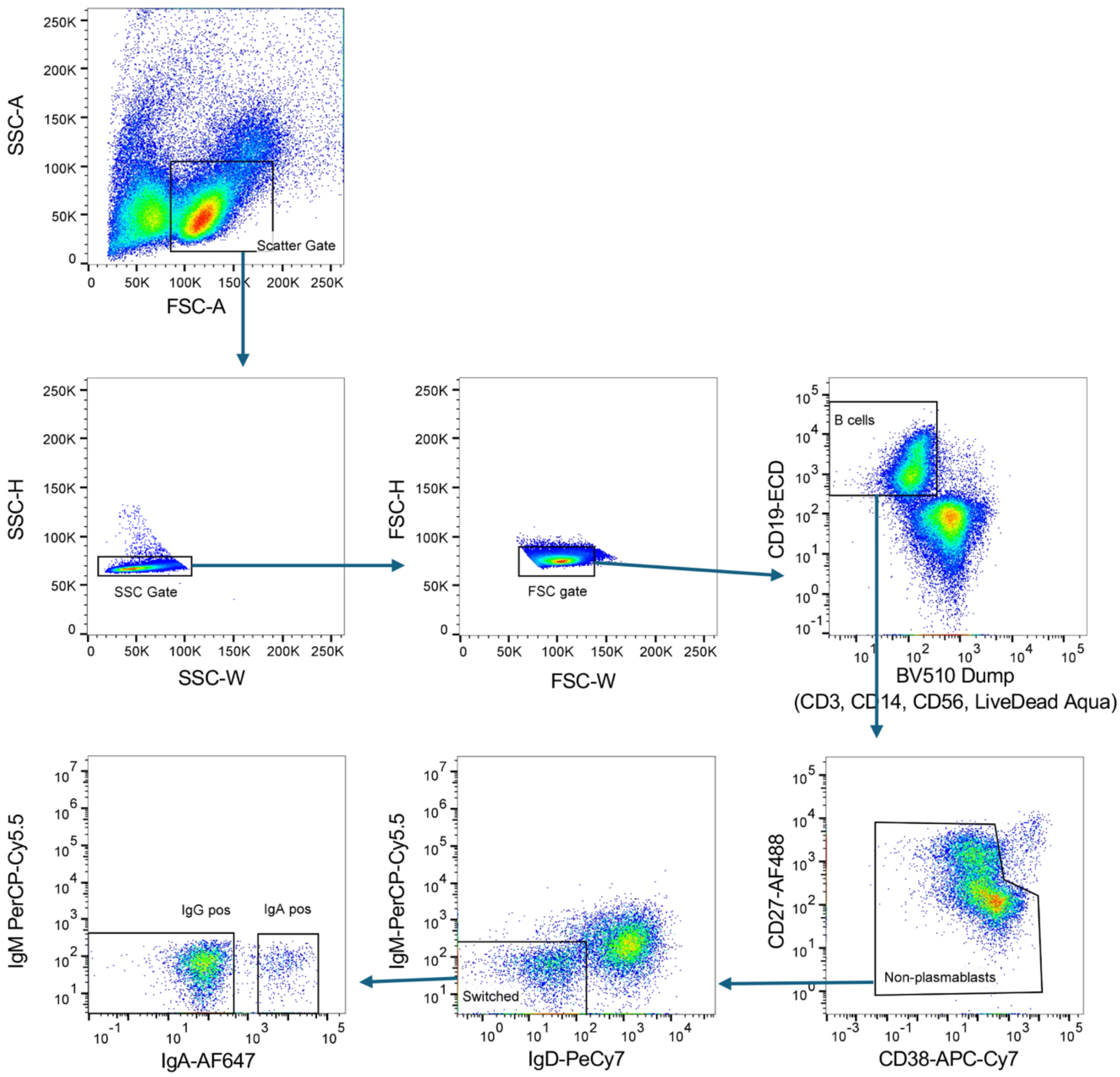

Supplementary Fig. 1: Gating strategy employed for FACS sorting of PBMCs for IgG+ B cells.

This gating strategy was used to isolate B cells for which supernatants were screened, as depicted in Figure 1c.

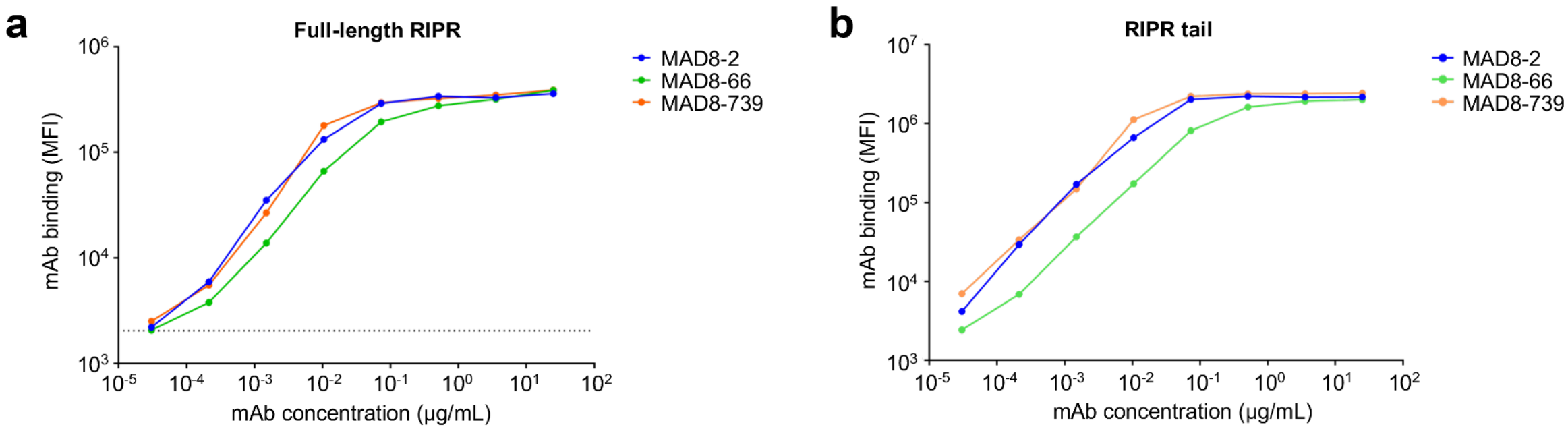

c

	MAD8-2	MAD8-66	MAD8-739
Isotype	IgG1	IgG1	IgG1
Heavy chain V-Gene	IGHV3-7*01	IGHV1-46*02	IGHV4-4*10
% V identity	89.58	86.11	90.97
Heavy chain D-Gene	IGHD3-22*01	IGHD5-12*01	IGHD6-19*01
Heavy chain J-Gene	IGHJ5*02 or IGHJ5*04	IGHJ3*01 or IGHJ3*02	IGHJ4*02
Light chain V-Gene	IGKV1-5*03	IGKV4-1*03	IGLV1-40*01 or IGLV1-40*02
% V identity	95.34	96.30	94.44
Light chain J-Gene	IGKJ2*03	IGKJ1*01	IGLJ2*01 or IGLJ2A*01 or IGLJ3*01 or IGLJ3*02

Supplementary Fig. 2: PfRIPR-specific human mAbs isolated from malaria-exposed individuals.

a, Binding of mAbs MAD8-2, MAD8-66 and MAD8-739 to PfRIPR-coated beads. **b,** Binding of mAbs MAD8-2, MAD8-66 and MAD8-739 to PfRIPR tail-coated beads. **c,** Gene and isotype information for MAD8-2, MAD8-66 and MAD8-739. Gene information was obtained from the International Immunogenetics Information System (IMGT) database.

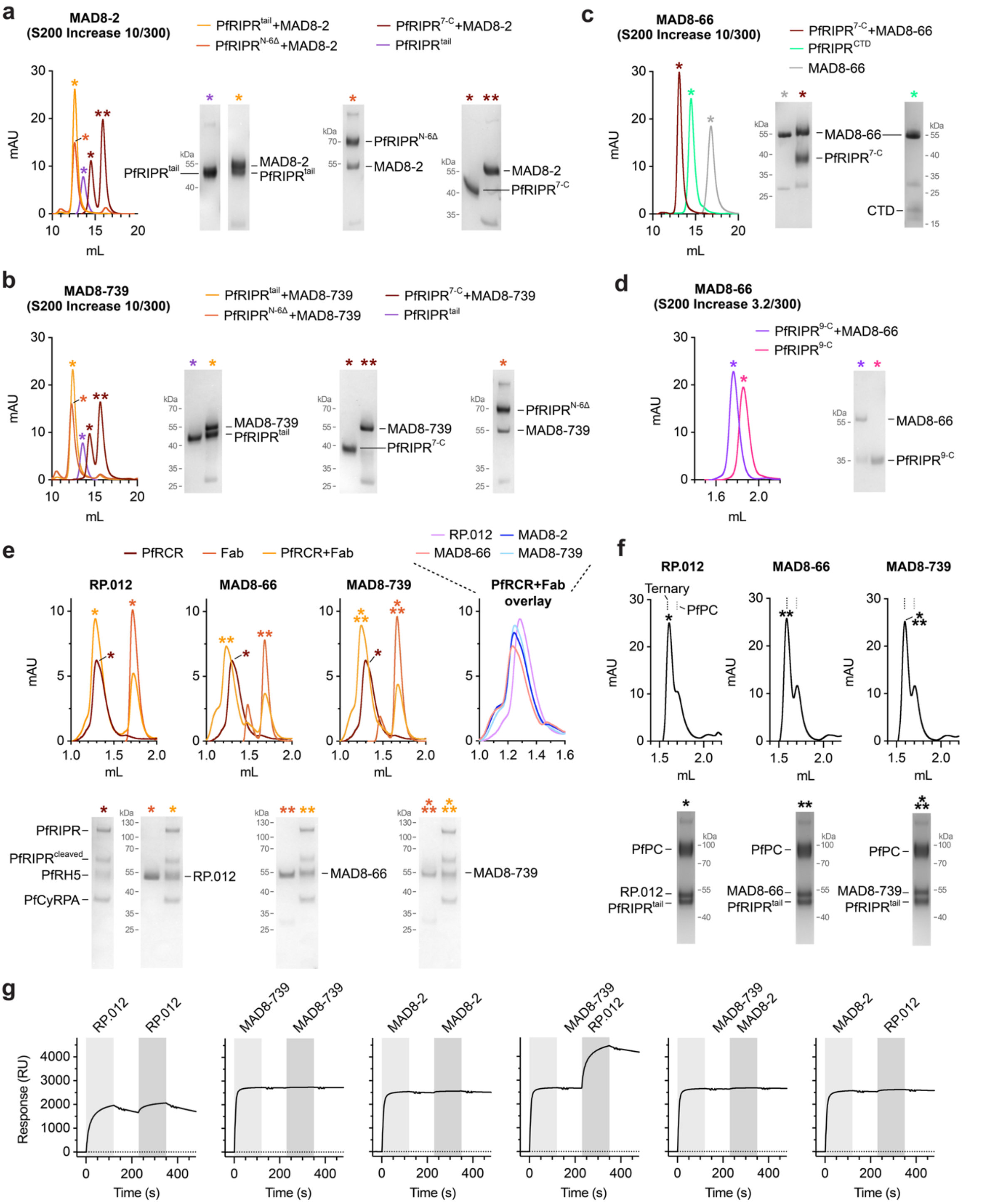

Supplementary Fig. 3: Epitope binning and competition analysis of PfRIPR antibodies.

a-d, SEC traces of MAD8-2, MAD8-66 and MAD8-739 Fab fragments with and without various fragments of PfRIPR to identify their epitopes, as summarised in Fig. 1f; Fab fragment (grey), PfRIPR^{tail} (purple), PfRIPR^{tail}+Fab (orange), PfRIPR$^{N\text{-}6\Delta}$+Fab (red), PfRIPR$^{7\text{-}C}$+Fab (brown), PfRIPR^{CTD}+Fab (light green), PfRIPR$^{9\text{-}C}$ (pink), PfRIPR$^{9\text{-}C}$+Fab (purple). For each analysis, peak fractions as identified by coloured asterisks were analysed by SDS-PAGE to verify co-elution of components. Each analysis was performed once. **e**, SEC traces of RP.012, MAD8-66 and MAD8-739 Fab fragments (red), PfRCR (brown) and their mixture (orange), illustrating co-elution of all Fabs with the intact PfRCR complex. Peak fractions, identified by the coloured asterisks, were analysed by SDS-PAGE to confirm co-elution of components. PfRIPR$^{67L(cleaved)}$ corresponds to PfRIPR cleaved in the L1-L2 loop generating the N- and C-halves of PfRIPR as described previously[2,8,65]. The

PfRCR trace and SDS-PAGE gel lane is replicated from Fig. 1g for comparisons in the presence of each Fab fragment. Adjacent right, overlay of traces for PfRCR + Fab fragments of RP.012 (pink), MAD8-2 (dark blue), MAD8-66 (salmon) and MAD8-739 (light blue), illustrating later elution of the PfRCR+RP.012 complex. Each analysis was performed once. **f**, SEC traces of RP.012, MAD8-66 or MAD8-739 Fab fragments after incubation with PfPTRAMP-PfCSS and PfRIPR tail. The dotted lines indicate the elution volume of the ternary complex peak observed for MAD8-2 (black) and PfPTRAMP-PfCSS (grey) as shown in Fig. 1h, illustrating that each antibody forms a ternary complex that elutes similarly, although the RP.012 complex elutes later. The fraction at this location was analysed by SDS-PAGE to verify co-elution of all three components. Each analysis was performed once. **g**, Representative raw SPR traces showing sequential injection of Fab fragments of EGF5-8 binding antibodies over PfRIPR immobilised on an SPR chip, demonstrating binding competition; n=3 independent technical replicates, 1 shown. Source data for panels in this figure are provided as a Source Data file.

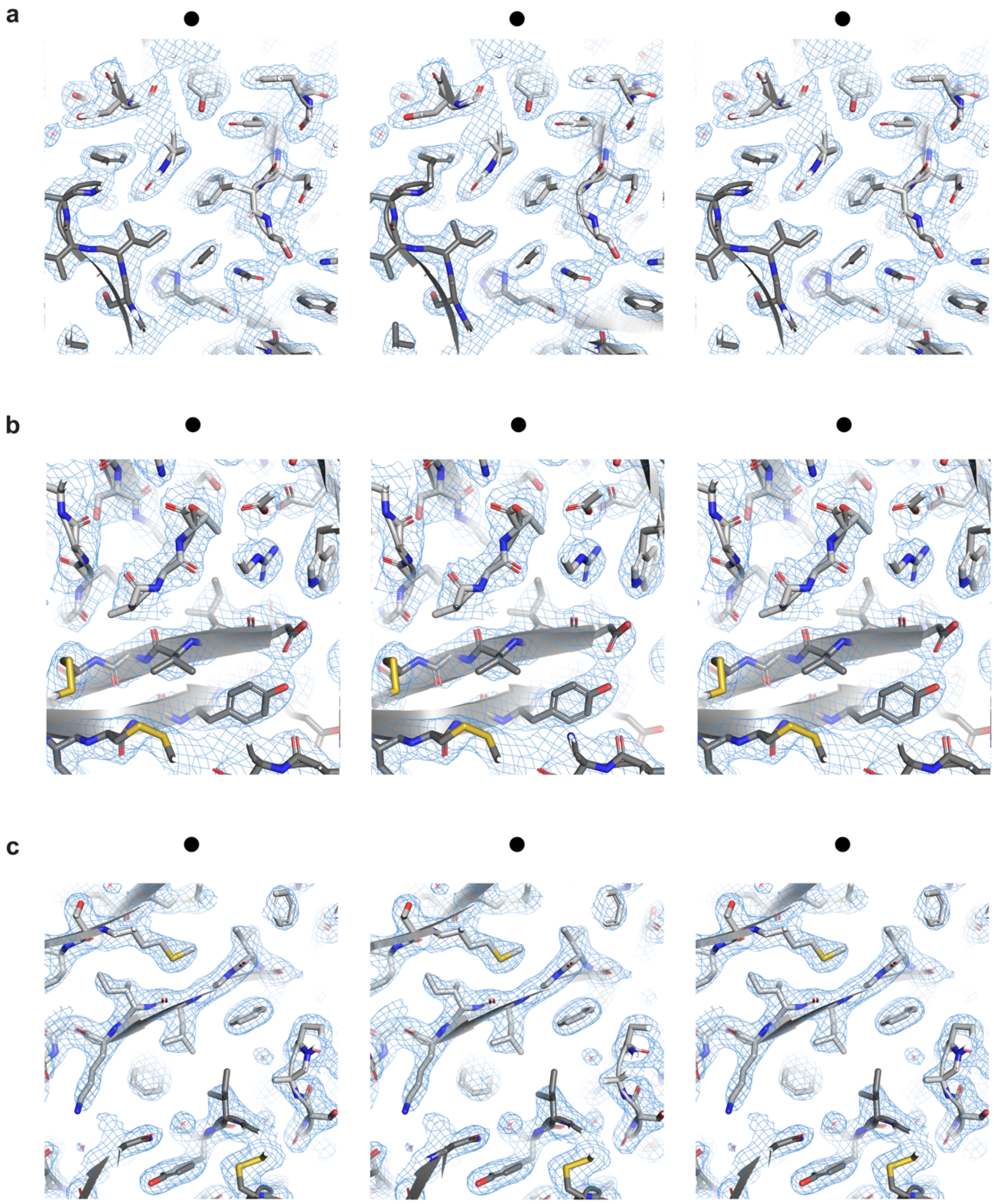

Supplementary Fig. 4: Quality of X-ray crystallography data

a-c, Cross-eye stereo views of X-ray crystallography electron density maps and their built models for each crystal structure in this manuscript (**a** = RP.012 and PfRIPR EGF6-7, **b** = MAD8-739 and PfRIPR EGF5-6, and **c** = MAD8-66 and PfRIPR EGF9-CTD), showing the interfaces of each antibody with PfRIPR. In each case, 2Fo-Fc maps are shown at a contour level of 1 sigma, shown as light blue isomesh. Structural models are shown in stick representation with PfRIPR in dark grey and antibody fragments in dark grey.

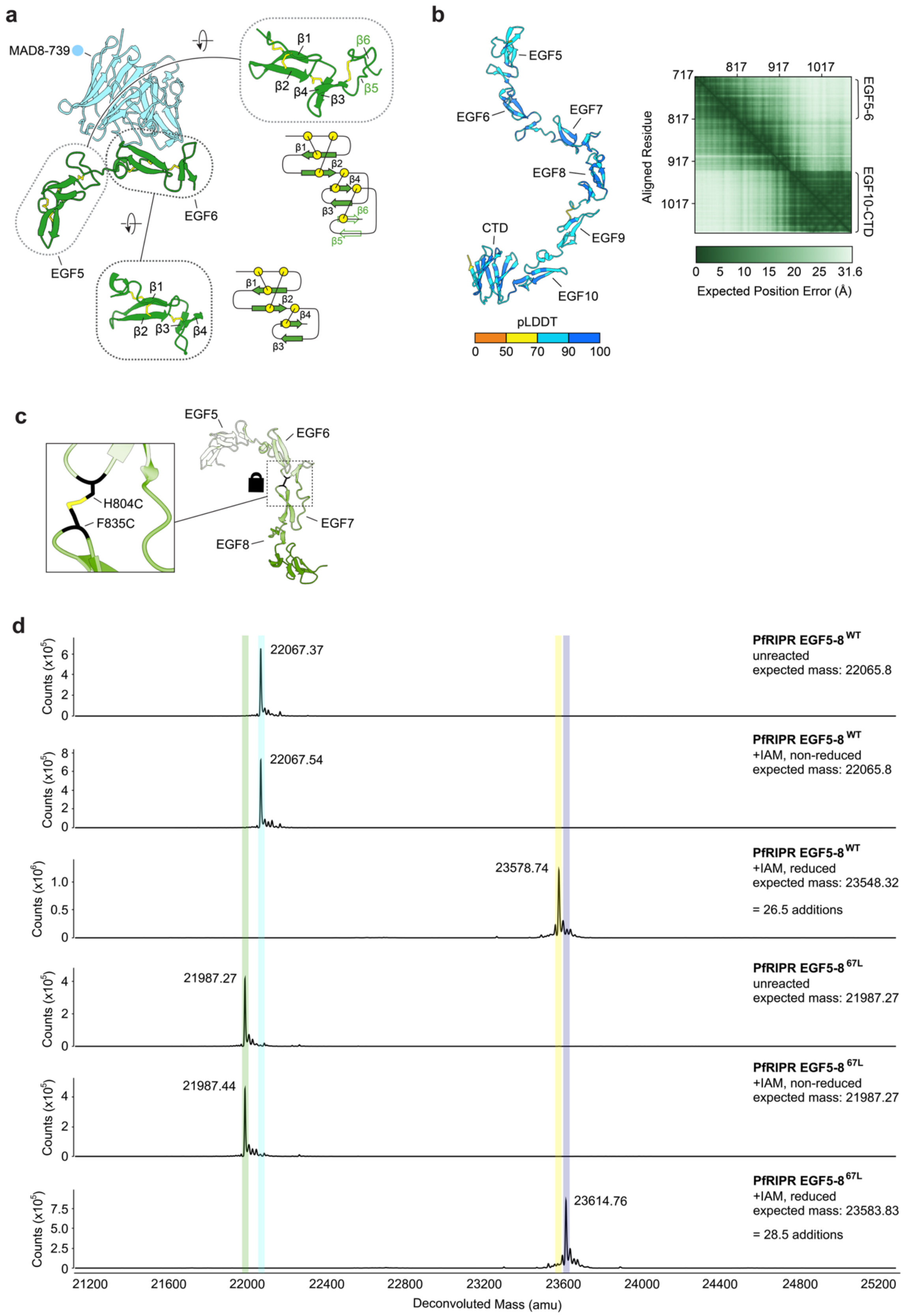

Supplementary Fig. 5: Structural analysis of PfRIPR and design of a lock between PfRIPR EGF6-7.

a, Structure of MAD8-739 (light blue) and PfRIPR EGF-like domains 5-6 (green) in cartoon representation with disulphide bonds shown as yellow sticks. Each domain is expanded and shown inset, to display these disulphide

bonds more clearly, with the major (β1-β2) and minor sheets (β3-β4) of the EGF-like fold labelled. EGF-like domain 5 contains an additional disulphide bond, and an extra structural element like an additional beta sheet (β5-β6), like a laminin fold[28]. A schematic representation of each EGF-like domain is shown adjacent to each inset. **b**, AlphaFold prediction of PfRIPR^{tail} in cartoon representation coloured by pLDDT (left) and its PAE matrix (right) with domains of interest labelled. **c**, Design of a cysteine-locked PfRIPR through introduction of a disulphide bond between EGF6 and 7 at H804 and F835 (as shown inset). **d**, Intact mass analysis of wild-type (EGF5-8^{WT}) or EGF6-7 cysteine-locked PfRIPR (EGF5-8^{67L}), either unreacted, or following reaction with iodoacetamide (IAM) under denaturing conditions both with and without reducing agent. The expected and detected deconvoluted masses are shown with bars highlighting masses observed for clarity. PfRIPR EGF5-8^{WT} and PfRIPR EGF5-8^{67L} match their expected unlabelled masses in the absence of reducing agent (green and blue bars, respectively), indicating that they each contain no free cysteine residues. Under reducing conditions, EGF5-8^{WT} is labelled with 26.5 carbamidomethyl groups indicating that it contains 13 disulphide bonds (yellow bar), and EGF5-8^{67L} is labelled with 28.5 carbamidomethyl groups indicating that it contains 14 disulphide bonds (13 native and 1 introduced, purple bar). Analysis was performed for each sample once. Source data for panels in this figure are provided as a Source Data file.

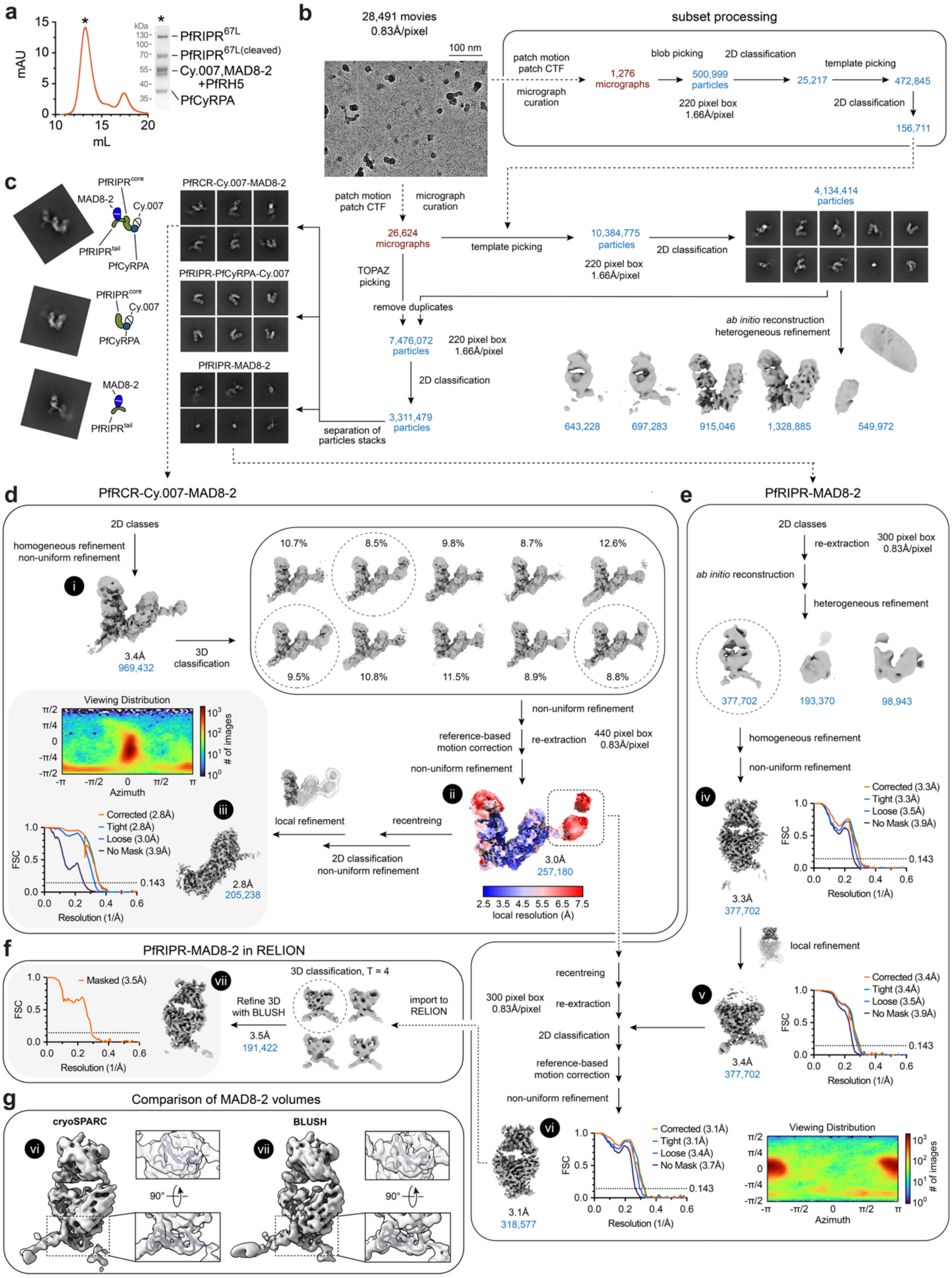

Supplementary Fig. 6: Structure determination of MAD8-2-bound PfRIPR.

a, Size exclusion chromatography trace showing isolation of a MAD8-2- and Cy.007-bound PfRCR complex with the peak fraction analysed by SDS-PAGE shown inset. PfRIPR$^{67L(cleaved)}$ corresponds to PfRIPR cleaved in the L1-L2 loop generating the N- and C-halves of PfRIPR as described previously[2,8,65]. Source data for are provided as a Source Data file. **b-f**) Processing pipeline of single particle cryo-EM data. A subset of micrographs was first processed to obtain templates for particle picking of the full dataset (panel b). Early processing indicated that the

cleaned particle stack was heterogeneous with three distinct species observed (panel c). These corresponded to the MAD8-2-bound PfRCR-Cy.007 complex, and this complex appearing as two 'fragments' of PfRIPR-PfCyPRA-Cy.007 and PfRIPR-MAD8-2. Example representative 2D class averages are provided with labelled schematic illustrations adjacent to demonstrate species identification (panel c). Particle stacks for PfRCR-Cy.007-MAD8-2 and PfRIPR-MAD8-2 were separated for processing separately (panels d-e) but were partially recombined later following particle recentering to help overcome particle orientation bias of the MAD8-2-bound PfRIPR volume. The final 3D refinement of PfRIPR-MAD8-2 was performed in RELION using BLUSH regularisation[62] leading to a visibly more isotropic volume (panel f). All cryo-EM maps referred to in the main text or methods are labelled with black circles to aid navigation (maps i-vii). Final maps for MAD8-2-bound PfRIPR-PfCyRPA-Cy.007 (map iii) and MAD8-2-bound PfRIPR (map vii) and their FSC curves are highlighted with grey boxes. **g**, Comparison of MAD8-2-bound PfRIPR volumes from cryoSPARC (map vi) and BLUSH (map vii) refinements, demonstrating improved visual quality but equivalent structural interpretations. Unsharpened volumes are shown with EGF6 of our refined model docked into each volume shown inset.

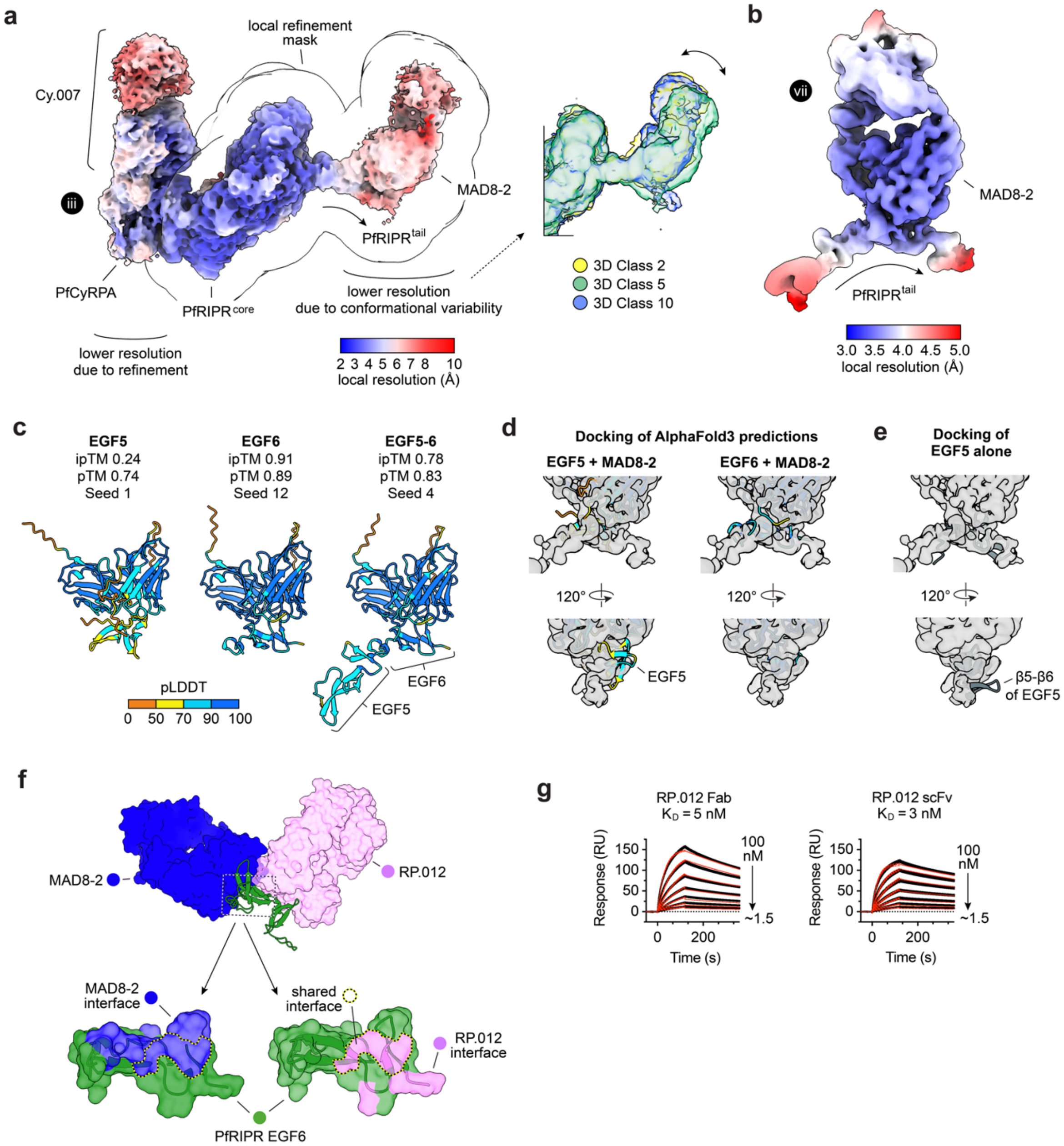

Supplementary Fig. 7: Cryo-EM volume interpretation and structural analyses.

a, Unsharpened cryo-EM volume (map iii) of MAD8.2-bound PfRIPR-PfCyRPA-Cy.007 coloured by estimated local resolution, demonstrating variable map resolution due to both local refinement (e.g. at PfCyRPA and Cy.007) and intrinsic conformational variability (e.g. at PfRIPR tail and MAD8-2). The mask used for local refinement of this volume is shown as a transparent surface. Inset right, overlay of 3 volumes from 3D classification (yellow, green, and blue transparent surfaces), revealing motion in PfRIPR tail that correlates with lower local resolution. **b**, Unsharpened cryo-EM volume (map vii) of MAD8-2 bound PfRIPR coloured by estimated local resolution. **c**, Top-ranking AlphaFold3 predicted models of MAD8-2 and PfRIPR EGF5, EGF6 or EGF5-6, with their seed number and ipTM and pTM scores (see also Supplementary Table 7). Models are shown as cartoons and coloured by pLDDT score. **d**, Docking of the top-ranking AlphaFold3 models of MAD8-2 and PfRIPR EGF5 or EGF6 into the unsharpened cryo-EM volume of MAD8-2 bound PfRIPR (map vii, semi-transparent surface), demonstrating that only the latter is consistent with the volume. Models are shown as cartoons, coloured by pLDDT score as in (c). **e**, Docking of EGF-like domain 5 of PfRIPR (from our MAD8-739-PfRIPR crystal structure detailed herein) into the unsharpened cryo-EM volume of MAD8-2-bound PfRIPR (map vii), demonstrating lack of density attributable to β5-β6 of EGF5, and therefore poor fit to the volume. **f**, Structural alignment of MAD8-2 and RP.012-bound PfRIPR demonstrating steric clash between these antibodies consistent with binding competition

data. PfRIPR (green) is shown in cartoon representation, MAD8-2 (dark blue) in surface representation, and RP.012 (pink) in transparent surface representation. Inset below, EGF6 of PfRIPR from each structure shown in cartoon and surface representation with the interaction surface of MAD8-2 (dark blue) and RP.012 (pink) highlighted, demonstrating overlap of binding interfaces with PfRIPR (outlined with a black and yellow border). **g,** SPR traces measuring the interaction of RP.012 Fab and scFv fragments with PfRIPR (black lines) and their kinetic fits (red), and their measured affinity stated above, using a two-fold dilution series starting at 100 nM antibody fragment (n=3 independent technical replicates); see also Supplementary Table 8. Source data for panels in this figure are provided as a Source Data file.

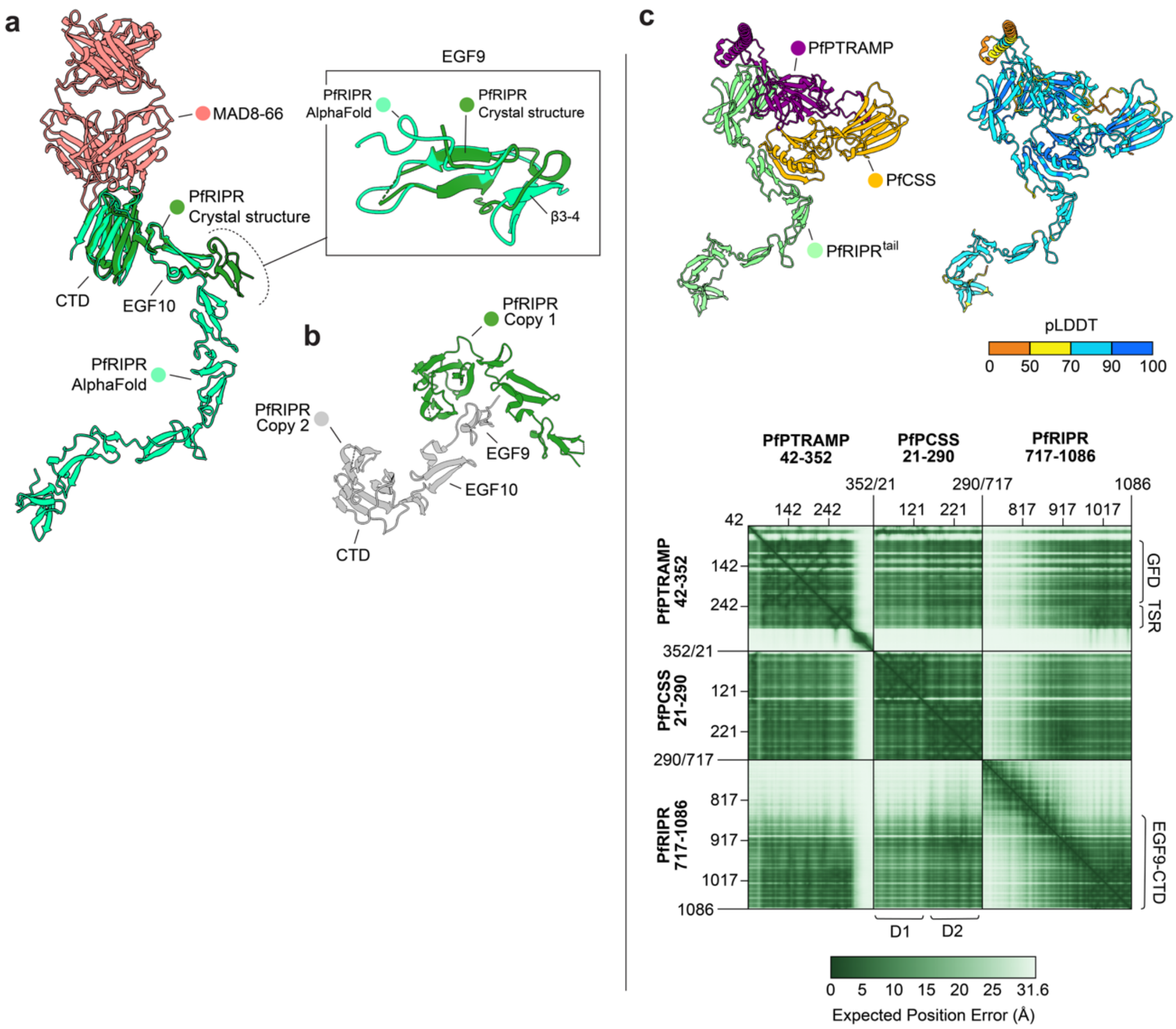

Supplementary Fig. 8: Analysis of PfRIPR-antibody structures.

a, Alignment of the crystal structure of MAD8-66 (pink) and PfRIPR EGF9-CTD (green) and the AlphaFold prediction of PfRIPR^{tail} (light green), illustrating different placements of EGF9 and a different conformation in β3-β4 of EGF9 (shown inset). **b**, Symmetry mates of PfRIPR EGF9-CTD (green and grey) illustrating that EGF9 forms crystal contacts with the CTD of its symmetry mate, likely inducing conformational changes in EGF9 (Fig. 3b and Supplementary Fig. 7a). **c**, AlphaFold3 prediction of PfPTRAMP-PfCSS-PfRIPR^{tail} in cartoon representation coloured by chain (left) and by pLDDT (right), and its PAE matrix (below) with domains of interest labelled.

Supplementary Table 1: Kinetic and affinity parameters for mAb binding to PfRIPR.

mAb	n	k_a ($M^{-1}s^{-1}$)	SE (k_a)	k_d (s^{-1})	SE (k_d)	K_D (M)	Chi^2 (RU^2)	U-value
RP.012	1	7.57×10^{4}	3.1×10^{2}	3.98×10^{-4}	6.1×10^{-6}	5.3×10^{-9}	1.37	2
	2	8.23×10^{4}	3.7×10^{2}	3.92×10^{-4}	7.2×10^{-6}	4.8×10^{-9}	1.78	3
MAD8-2	1	7.58×10^{5}	4.0×10^{3}	1.15×10^{-4}	4.7×10^{-6}	1.5×10^{-10}	2.09	9
	2	7.35×10^{5}	7.7×10^{2}	1.43×10^{-4}	3.6×10^{-6}	1.9×10^{-10}	1.16	5
MAD8-66	1	1.15×10^{6}	6.7×10^{3}	1.06×10^{-4}	4.8×10^{-6}	9.2×10^{-11}	9.40	9
	2	1.21×10^{6}	3.3×10^{3}	1.09×10^{-4}	2.3×10^{-6}	9.0×10^{-11}	1.70	4
MAD8-739	1	4.27×10^{5}	8.8×10^{2}	7.02×10^{-5}	1.8×10^{-6}	1.6×10^{-10}	1.31	7
	2	4.43×10^{5}	1.5×10^{3}	8.17×10^{-5}	1.8×10^{-6}	1.9×10^{-10}	0.75	4

Supplementary Table 2: X-ray crystallography data collection and refinement statistics.

	**RP.012-bound PfRIPR**<br>PDB ID 9TUW	**MAD8-66-bound PfRIPR**<br>PDB ID 9TUX	**MAD8-739-bound PfRIPR**<br>PDB ID 9TUY
**Data Collection**			
Space group	P 1	P 21 21 21	P 3 1
Cell dimensions			
a, b, c (Å)	50.16, 50.98, 109.78	52.79, 63.24, 250.73	89.64, 89.64, 141.34
α, β, γ (°)	76.82, 83.26, 76.50	90.00, 90.00, 90.00	90.00, 90.00, 120.00
Wavelength	0.9537 Å	0.9537 Å	0.6199 Å
Resolution (Å)	48.67-2.20 (2.23-2.20)	63.25-2.25 (2.29-2.25)	42.72-2.80 (2.95-2.80)
Total Observations	186049 (9015)	549427 (28167)	335117 (49712)
Total Unique	51504 (2453)	41042 (2058)	31298 (4549)
R_{merge} (%)	10.1 (132.7)	29.4 (410.8)	32.6 (379.2)
R_{meas} (%)	11.9 (155.5)	30.6 (426.6)	34.3 (397.8)
R_{pim} (%)	6.2 (80.6)	8.3 (41.5)	10.5 (120.2)
$CC_{1/2}$	0.995 (0.317)	0.996 (0.415)	0.993 (0.810)
$I/\sigma(I)$	11.1 (0.8)	8.3 (0.7)	5.6 (1.1)
Completeness (%)	98.3 (94.3)	100.0 (100.0)	100.0 (100.0)
Multiplicity	3.6 (3.7)	13.4 (13.7)	10.7 (10.9)
Wilson B factor	53.41	47.08	68.7
**Refinement**			
Reflections	51362	40916	31142
Rwork / Rfree (%)	24.1/25.6	23.0/26.2	22.8/25.5
Average B factor			
Protein	65.67	56.58	85.10
Water	55.29	54.16	71.16
Number of residues			
Protein	959	605	674
Water	157	93	63
RMSDs			
Bond lengths (Å)	0.010	0.003	0.010
Bond angles (°)	1.40	0.58	1.42
Ramachandran plot			
Favored (%)	98.0	98.0	94.4
Allowed (%)	2.0	2.0	5.6
Outliers (%)	0	0	0
Clashscore	5.36	2.80	3.00
MolProbity score	1.82	1.07	1.72

Supplementary Table 3: Interaction contacts between RP.012 and PfRIPR.

PfRIPR				RP.012				
**Chain**	**Residue**	**Group**	**Location**	**Chain**	**Residue**	**Group**	**Location**	**Interaction**
D	E800	Side chain	EGF6	F	S87	Side chain	Kappa FR3	H-bond
D	E803	Side chain	EGF6	F	S70	Side chain	CDR L2	H-bond
D	E803	Backbone	EGF6	F	T51	Side chain	CDR L1	H-bond
D	Y806	Side chain	EGF6	F	R113	Side chain	CDR L3	Cation-pi
D	R807	Backbone	EGF6	F	N48	Side chain	CDR L1	H-bond
D	N816	Side chain	EGF6	F	S112	Side chain	CDR L3	H-bond
D	D817	Backbone	EGF6	F	S112	Backbone	CDR L3	H-bond
	D817	Side chain	EGF7	F	Y114	Backbone	CDR L3	H-bond
				F	L66	Side chain	Kappa FR2	Hydrophobic
				F	Y69	Side chain	Kappa FR2	Hydrophobic
				F	Y111	Side chain	CDR L3	Hydrophobic
				F	F116	Side chain	CDR L3	Hydrophobic
D	Y818	Side chain	EGF7					Hydrophobic
D	D821	Side chain	EGF7	E	R70	Side chain	Heavy FR2	Salt bridge
	D821	Side chain	EGF7	E	K79	Side chain	Heavy FR3	Salt bridge
	D821	Side chain	EGF7	F	Y114	Side chain	Heavy FR3	H-bond
	D821	Side chain	EGF7	E	R70	Side chain	Heavy FR2	H-bond
D	I832	Side chain	EGF7					Hydrophobic
D	N834	Backbone	EGF7	E	Y52	Side chain	CDR H1	H-bond
D	N834	Side chain	EGF7	E	Y52	Side chain	CDR H1	H-bond
D	F835	Backbone	EGF7	E	L120	Backbone	CDR H3	H-bond
	F835	Side chain	EGF7	E	D123	Side chain	CDR H3	Anion-pi
D	F835	Side chain	EGF7					Hydrophobic
D	P837	Side chain	EGF7					Hydrophobic
				E	W53	Side chain	CDR H1	Hydrophobic
				E	L120	Side chain	CDR H3	Hydrophobic

Supplementary Table 4: Interaction contacts between MAD8-739 and PfRIPR.

PfRIPR				MAD8-739				
**Chain**	**Residue**	**Group**	**Location**	**Chain**	**Residue**	**Group**	**Location**	**Interaction**
B	K759	Side chain	EGF5	A	Y84	Side chain	CDR H2	H-bond
B	E761	Side chain	EGF5	A	H85	Side chain	CDR H2	H-bond
	E761	Side chain	EGF5	A	Y84	Side chain	CDR H2	H-bond
	E761	Side chain	EGF5	A	H85	Side chain	CDR H2	Salt bridge
B	R774	Side chain	EGF6	A	D63	Side chain	CDR H1	H-bond
	R774	Side chain	EGF6	A	D63	Side chain	CDR H1	Salt bridge
B	Y777	Side chain	EGF6					Hydrophobic
B	I780	Side chain	EGF6					Hydrophobic
B	E792	Backbone	EGF6	A	K88	Side chain	CDR H2	H-bond
B	E793	Backbone	EGF6	A	K88	Side chain	CDR H2	H-bond
B	T794	Backbone	EGF6	A	R131	Side chain	CDR H3	H-bond
B	D795	Backbone	EGF6	A	R131	Side chain	CDR H3	H-bond
	D795	Side chain	EGF6	A	W65	Side chain	CDR H1	H-bond
B	I796	Side chain	EGF6					Hydrophobic
B	V797	Backbone	EGF6	A	G137	Backbone	CDR H3	H-bond
B	K798	Side chain	EGF6	A	D268	Side chain	CDR L3	H-bond
	K798	Side chain	EGF6	A	G204	Backbone	CDR L1	H-bond
	K798	Side chain	EGF6	A	Y206	Side chain	CDR L1	H-bond
	K798	Side chain	EGF6	A	D268	Side chain	CDR L3	Salt bridge
B	C799	Backbone	EGF6	A	G205	Backbone	CDR L1	H-bond
B	R810	Backbone	EGF6	A	T244	Backbone	LambdaFR3	H-bond
B	E812	Side chain	EGF6	A	S242	Side chain	LambdaFR3	H-bond
				A	P134	Side chain	CDR H3	Hydrophobic
B	R810	Backbone	EGF6	A	T244	Backbone	LambdaFR3	H-bond
				A	Y266	Side chain	CDR L3	Hydrophobic

Supplementary Table 5: Kinetic and affinity parameters for mAb binding to PfRIPR^{67L}.

mAb	n	k_a ($M^{-1}s^{-1}$)	SE (k_a)	k_d (s^{-1})	SE (k_d)	K_D (M)	Chi^2 (RU^2)	U-value
RP.012	1	n/a	n/a	n/a	n/a	n/a	n/a	n/a
	2	n/a	n/a	n/a	n/a	n/a	n/a	n/a
MAD8-2	1	$8.66x10^5$	$2.8x10^3$	$1.14x10^{-4}$	$2.8x10^{-6}$	$1.3x10^{-10}$	0.80	4
	2	$8.99x10^5$	$3.8x10^3$	$1.26x10^{-4}$	$3.6x10^{-6}$	$1.4x10^{-10}$	1.16	7

Supplementary Table 6: Cryo-EM data collection and refinement statistics.

	**MAD8-2-bound PfRIPR**<br>EMDB-56281<br>PDB ID 9TUZ	**PfRIPR-PfCyRPA**<br>EMDB-56282<br>PDB ID 9TV0
**Data collection and processing**		
Microscope	Titan Krios	Titan Krios
Detector	Gatan BioQuantum K3	Gatan BioQuantum K3
Energy Filter	20 eV	20 eV
Magnification	58,149	58,149
Voltage (kV)	300	300
Electron exposure ($e^{-}/Å^2$)	39.7	39.7
Defocus range (μm)	-1.2 to -2.6	-1.2 to -2.6
Pixel size (Å)	0.83	0.83
Symmetry imposed	C1	C1
Initial particles images (no.)	7,476,072	7,476,072
Final particle images (no.)	191,422	205,238
Map resolution (Å)	3.5	2.8
FSC threshold	0.143	0.143
Map resolution range (Å)	3.3-6.7	2.5-15.0
FSC threshold	0.143	0.143
Map sharpening B-factor ($Å^2$)	-104.4	-87.1, DeepEMhancer[66]
**Refinement**		
Initial model used (PDB code)	AlphaFold3	8CDD, 7PHV, AlphaFold2
Model resolution (Å)	4.2	3.4
FSC threshold	0.5	0.5
Model composition		
Non-hydrogen atoms	3693	7503
Protein residues	482	919
Ligands	n/a	n/a
B factors ($Å^2$)		
Protein	94.0	68.44
Ligands	n/a	n/a
R.m.s deviations		
Bond length (Å)	0.003	0.004
Bond angles (°)	0.513	0.526
Validation		
Molprobity score	1.43	1.59
Clash score	5.93	4.63
Poor rotamers (%)	0.48	1.04
Ramachandran plot		
Favored (%)	97.48	95.17
Allowed (%)	2.52	4.83
Outliers (%)	0	0
Model vs. data fit		
CC (mask)	0.66	0.74
CC (box)	0.82	0.79

Supplementary Table 7: Scores for AlphaFold3 predicted models of PfRIPR and MAD8-2.

Domain	Rank	Seed	ipTM	pTM		Domain	Rank	Seed	ipTM	pTM
EGF5	1	1	0.24	0.74		EGF6	1	12	0.91	0.89
EGF5	2	2	0.23	0.74		EGF6	2	19	0.91	0.88
EGF5	3	4	0.23	0.73		EGF6	3	5	0.90	0.88
EGF5	4	6	0.22	0.76		EGF6	4	6	0.90	0.88
EGF5	5	7	0.22	0.73		EGF6	5	7	0.90	0.88
EGF5	6	3	0.21	0.73		EGF6	6	9	0.90	0.87
EGF5	7	8	0.21	0.74		EGF6	7	11	0.90	0.89
EGF5	8	9	0.21	0.72		EGF6	8	17	0.90	0.88
EGF5	9	10	0.21	0.74		EGF6	9	20	0.90	0.89
EGF5	10	5	0.20	0.74		EGF6	10	3	0.89	0.89
EGF5-6	1	4	0.78	0.83		EGF6	11	10	0.89	0.88
EGF5-6	2	10	0.77	0.82		EGF6	12	18	0.89	0.88
EGF5-6	3	8	0.32	0.67		EGF6	13	13	0.88	0.87
EGF5-6	4	2	0.29	0.67		EGF6	14	14	0.61	0.80
EGF5-6	5	7	0.27	0.66		EGF6	15	16	0.60	0.80
EGF5-6	6	5	0.24	0.65		EGF6	16	1	0.55	0.79
EGF5-6	7	1	0.22	0.64		EGF6	17	8	0.53	0.79
EGF5-6	8	3	0.17	0.64		EGF6	18	4	0.50	0.78
EGF5-6	9	6	0.13	0.63		EGF6	19	2	0.44	0.77
EGF5-6	10	9	0.10	0.62		EGF6	20	15	0.37	0.77

Supplementary Table 8: Kinetic and affinity parameters for RP.012 fragments binding to PfRIPR.

Fragment	n	k_a ($M^{-1}s^{-1}$)	SE (k_a)	k_d (s^{-1})	SE (k_d)	K_D (M)	Chi^2 (RU^2)	U-value
RP.012 Fab	1-3	3.10×10^5	2.8×10^2	1.53×10^{-3}	2.5×10^{-6}	4.9×10^{-9}	14	2
RP.012 scFv	1-3	3.97×10^5	3.2×10^2	1.13×10^{-3}	2.3×10^{-6}	2.9×10^{-9}	9.4	2

Supplementary Table 9: Interaction contacts between MAD8-66 and PfRIPR.

PfRIPR				MAD8-66				
Chain	Residue	Group	Location	Chain	Residue	Group	Location	Interaction
C	E983	Side chain	EGF10-CTD	B	N54	Side chain	CDR H2	H-bond
C	Y985	Side chain	EGF10-CTD	B	D28	Side chain	CDR H1	H-bond
C	I1000	Side chain	CTD					Hydrophobic
C	A1003	Side chain	CTD					Hydrophobic
C	N1045	Backbone	CTD	B	K52	Side chain	CDR H2	H-bond
C	E1046	Side chain	CTD	B	N57	Side chain	CDR H2	H-bond
	E1046	Side chain	CTD	B	K52	Side chain	CDR H2	Salt bridge
C	I1047	Side chain	CTD					Hydrophobic
C	F1048	Side chain	CTD					Hydrophobic
C	T1073	Backbone	CTD	A	S99	Side chain	CDR L3	H-bond
	T1073	Backbone	CTD	A	A100	Backbone	CDR L3	H-bond
C	C1074	Side chain	CTD	A	S98	Backbone	CDR L3	H-bond
C	I1075	Side chain	CTD					Hydrophobic
C	E1077	Backbone	CTD	B	D104	Side chain	CDR H3	H-bond
	E1077	Side chain	CTD	B	F33	Backbone	CDR H1	H-bond
	E1077	Side chain	CTD	B	H35	Side chain	Heavy FR2	Salt bridge
C	N1078	Backbone	CTD	B	D104	Side chain	CDR H3	H-bond
	N1078	Side chain	CTD	B	D104	Side chain	CDR H3	H-bond
	N1078	Side chain	CTD	B	H100	Backbone	CDR H3	H-bond
C	F1080	Side chain	CTD	A	N34	Side chain	CDR L1	Polar-pi
				A	F31	Side chain	CDR L1	Hydrophobic
				A	A33	Side chain	CDR L1	Hydrophobic
				A	Y38	Side chain	CDR L1	Hydrophobic
				A	W56	Side chain	CDR L2	Hydrophobic
				A	A100	Side chain	CDR L3	Hydrophobic
				A	W102	Side chain	CDR L3	Hydrophobic
				B	F33	Side chain	CDR H1	Hydrophobic
				B	L50	Side chain	Heavy FR2	Hydrophobic
				B	A59	Side chain	Heavy FR3	Hydrophobic
				B	V99	Side chain	CDR H3	Hydrophobic
