# Supplementary material for: Dynamic hinge-motion of PfRIPR revealed by malaria invasion inhibitory antibodies
Source: Nat Commun. 2026 Aug 24;17:10727. doi: 10.1038/s41467-026-77069-z (PMC13658223; doi:10.1038/s41467-026-77069-z)
Supplement: Supplementary file 2 [file 41467_2026_77069_MOESM2_ESM.pdf]

Description of Additional Supplementary Files

File name: Supplementary Movie 1
Description: Conformational variability of PfRIPR tail. Morph of three volumes from 3D classification of PfRCR-Cy.007-MAD8-2 showing conformational variability of the tail of PfRIPR.
