# Supplementary material for: Dynamic hinge-motion of PfRIPR revealed by malaria invasion inhibitory antibodies
Source: Nat Commun. 2026 Aug 24;17:10727. doi: 10.1038/s41467-026-77069-z (PMC13658223; doi:10.1038/s41467-026-77069-z)
Supplement: Supplementary file 3 [file 41467_2026_77069_MOESM4_ESM.pdf]

nature portfolio

Corresponding author(s): Matt Higgins

Last updated by author(s): Jun 27, 2026

Reporting Summary

Nature Portfolio wishes to improve the reproducibility of the work that we publish. This form provides structure for consistency and transparency in reporting. For further information on Nature Portfolio policies, see our Editorial Policies and the Editorial Policy Checklist.

Statistics

For all statistical analyses, confirm that the following items are present in the figure legend, table legend, main text, or Methods section.

n/a	Confirmed	
☐	☒	The exact sample size (*n*) for each experimental group/condition, given as a discrete number and unit of measurement
☐	☒	A statement on whether measurements were taken from distinct samples or whether the same sample was measured repeatedly
☐	☒	The statistical test(s) used AND whether they are one- or two-sided<br>*Only common tests should be described solely by name; describe more complex techniques in the Methods section.*
☒	☐	A description of all covariates tested
☒	☐	A description of any assumptions or corrections, such as tests of normality and adjustment for multiple comparisons
☐	☒	A full description of the statistical parameters including central tendency (e.g. means) or other basic estimates (e.g. regression coefficient) AND variation (e.g. standard deviation) or associated estimates of uncertainty (e.g. confidence intervals)
☒	☐	For null hypothesis testing, the test statistic (e.g. *F*, *t*, *r*) with confidence intervals, effect sizes, degrees of freedom and *P* value noted<br>*Give P values as exact values whenever suitable.*
☒	☐	For Bayesian analysis, information on the choice of priors and Markov chain Monte Carlo settings
☒	☐	For hierarchical and complex designs, identification of the appropriate level for tests and full reporting of outcomes
☒	☐	Estimates of effect sizes (e.g. Cohen's *d*, Pearson's *r*), indicating how they were calculated

Our web collection on statistics for biologists contains articles on many of the points above.

Software and code

Policy information about availability of computer code

Data collection	Software used for data collection are commercially available or openly accessible. Cryo-EM data were collected using EPU version 2.9.0.1519 (Thermo Fisher). SPR data were acquired using T200 Biacore Software version 2.0 (GE Healthcare). Mass spectrometry data analysis was performed using Masshunter Qualitative Analysis v7.0 (Agilent Technologies).
Data analysis	Data analysis was performed as described in the methods section using commercially available or openly accessible software. Software used for cryo-EM data processing (CryoSPARC v4 and RELION 5.0.0) are standard and freely available to academic users. Model building and refinement was performed with COOT version 0.9.3, ISOLDE v1.5 and PHENIX v1.21.2. PHASER and AIMLESS of the CCP4 suite (v8.0.019), BUSTER (v2.10.4), PHENIX (v1.21.2), and COOT (v0.9.3) were used for X-ray crystallographic data processing and model building. A local installation of AlphaFold2 and the AlphaFold3 server were used for structure predictions. ChimeraX v1.8 was used for structure visualisation. BIAevaluation software v1.0 is provided with the Biacore T200 SPR system and is standard in the field. GraphPad Prism v9.3.1 and 10.6.1 was used to generate graphs and perform statistical analyses. Adobe Illustrator was used to generate figures.

For manuscripts utilizing custom algorithms or software that are central to the research but not yet described in published literature, software must be made available to editors and reviewers. We strongly encourage code deposition in a community repository (e.g. GitHub). See the Nature Portfolio guidelines for submitting code & software for further information.

Data

Policy information about availability of data

All manuscripts must include a data availability statement. This statement should provide the following information, where applicable:

- Accession codes, unique identifiers, or web links for publicly available datasets
- A description of any restrictions on data availability
- For clinical datasets or third party data, please ensure that the statement adheres to our policy

Crystallographic data and protein models are available from the Protein Data Bank (PDB) under accession codes 9TUW for RP.012-bound PfRIPR, 9TUX for MAD8-66-bound PfRIPR, and 9TUY for MAD8-739-bound PfRIPR. Cryo-EM maps and associated co-ordinates for MAD8.2-bound PfRIPR are available from the Electron Microscopy Data Bank (EMDB) under accession code 9TUZ and EMDB-56281, and for PfRIPR-PfCyRPA at 9TV0 and EMDB-56282. A previously published structure and cryo-EM map have been used in this study; these can be found in the PDB under accession codes 8CDD and the EMDB at EMDB-16640. All other data is available from the authors on request. Unique reagents generated in this study will be made available on request by the lead contact with a completed Materials Transfer Agreement.

Research involving human participants, their data, or biological material

Policy information about studies with human participants or human data. See also policy information about sex, gender (identity/presentation), and sexual orientation and race, ethnicity and racism.

Reporting on sex and gender	Not applicable as samples were anonymous and antibodies or data is not linked back to individual participants.
Reporting on race, ethnicity, or other socially relevant groupings	N/A
Population characteristics	N/A
Recruitment	N/A
Ethics oversight	The Kalifabougou cohort study is an ongoing longitudinal study approved by the Ethics Committee of the Faculty of Medicine, Pharmacy and Dentistry at the University of Sciences, Technique and Technology of Bamako, and the Institutional Review Board of the National Institute of Allergy and Infectious Diseases, National Institutes of Health (NIH IRB protocol number: 11IN126).

Note that full information on the approval of the study protocol must also be provided in the manuscript.

Field-specific reporting

Please select the one below that is the best fit for your research. If you are not sure, read the appropriate sections before making your selection.

☒ Life sciences ☐ Behavioural & social sciences ☐ Ecological, evolutionary & environmental sciences

For a reference copy of the document with all sections, see nature.com/documents/nr-reporting-summary-flat.pdf

Life sciences study design

All studies must disclose on these points even when the disclosure is negative.

Sample size	Sample sizes are described in figure legends and methods. No statistical method was used to predetermine sample size. Experiments were planned based on previous studies using equivalent methods and scoping experiments. Statistical tests were used to assess whether sample sizes were sufficient.
Data exclusions	For cryo-EM workflows particles not contributing to classes of interest were excluded from the final reconstructions as standard.
Replication	The number of repeats for each relevant experiment are given in figure legends and the methods. Typically, experiments were performed in independent technical triplicates.
Randomization	As is standard for cryo-EM workflows, particles were split randomly into two halves and refined separately, as is automatically implemented in CryoSPARC. Randomisation of other experiments was not relevant to this study as no subjective judgements were required about which data to include, exclude, or measure.
Blinding	The investigators were not blinded to the group allocation during the experiment and/or when assessing the outcome, as analysis were performed on quantitative endpoints that are not subject to investigator bias.

Reporting for specific materials, systems and methods

We require information from authors about some types of materials, experimental systems and methods used in many studies. Here, indicate whether each material, system or method listed is relevant to your study. If you are not sure if a list item applies to your research, read the appropriate section before selecting a response.

Materials & experimental systems

n/a	Involved in the study
☐	☒ Antibodies
☐	☒ Eukaryotic cell lines
☒	☐ Palaeontology and archaeology
☒	☐ Animals and other organisms
☒	☐ Clinical data
☒	☐ Dual use research of concern
☒	☐ Plants

Methods

n/a	Involved in the study
☒	☐ ChIP-seq
☒	☐ Flow cytometry
☒	☐ MRI-based neuroimaging

Antibodies

Antibodies used

Monoclonal antibodies (mAbs) RP.012 (murine anti-PfRIPR; Williams et al), MAD8-2, MAD8-66 and MAD8-739 (human anti-PfRIPR; this study) were characterised in this study. The monoclonal Cy.007 (chicken anti-PfCyRPA; Ragotte et al.) was used to aid structural analysis. Monoclonals 2AC7 (mouse anti-PfRH5; Douglas et al.), R5.034 (mouse anti-PfRH5; Barrett et al.), R5.016 (mouse anti-PfRH5; Alanine et al.), and DB8 (human anti-PvDBPII, Rawlinson et al.) were used as controls in growth inhibition assays. Antibodies CD3-BV510 (Cat. # 317332; RRID: AB_2561943), CD14-BV510 (Cat. # 301842; RRID: AB_2561946), CD56-BV510 (Cat. # 318340; RRID: AB_2561944), CD27-Alexa Fluor 488 (Cat. # 393204; RRID: AB_2750089), CD38-APC/Cy7 (Cat. # 303534; RRID: AB_2561605), CD19-ECD (Cat. # IM2708U; RRID: AB_130854), Goat anti-human IgG-Alexa Fluor 647 (Cat. # 109-606-170; RRID: AB_2337902), Goat anti-human IgA-Alexa Fluor 647 (Cat. # 109-606-011; RRID: AB_2337895), IgM-PerCP-Cy5.5 (Cat. # 561285; RRID: AB_10611998), IgD-PE-Cy7 (Cat. # 561314; RRID: AB_10642457), and 6x-His Tag Monoclonal Antibody (HIS.H8), Biotin (Cat. # MA1-21315-BTIN; RRID: AB_557403) were used during isolation of human antibodies.

Validation

Commercially available antibodies were validated by suppliers. Cy.007 (Ragotte et al.), 2AC7 (Douglas et al.), R5.034 (Barrett et al.), R5.016 (Alanine et al.) and DB8 (Rawlinson et al.) have been characterised previously by SPR and X-ray crystallography.

Eukaryotic cell lines

Policy information about cell lines and Sex and Gender in Research

Cell line source(s)

Commercially available Expi293F™ cells (ThermoFisher, cat: A14635), Expi293F™ GnTI- cells (Thermo Fisher, cat: A39240), FreeStyle™ 293-F cells (Thermo Fisher, cat: R79007), and Drosophila S2 cells (ExpreS2ion Biotechnologies) were used for recombinant protein expression.

Authentication

The cells were freshly obtained from commercial sources and were not validated.

Mycoplasma contamination

The cells were freshly obtained from commercial sources and were not validated.

Commonly misidentified lines
(See ICLAC register)

N/A

Plants

Seed stocks

N/A

Novel plant genotypes

N/A

Authentication

N/A
