# Supplementary material for: Dynamic hinge-motion of PfRIPR revealed by malaria invasion inhibitory antibodies
Source: Nat Commun. 2026 Aug 24;17:10727. doi: 10.1038/s41467-026-77069-z (PMC13658223; doi:10.1038/s41467-026-77069-z)
Supplement: Supplementary file 4 [file 41467_2026_77069_MOESM5_ESM.pdf]

nature portfolio

# Peer Review File

# Dynamic hinge-motion of PfRIPR revealed by malaria invasion inhibitory antibodies

Corresponding Author: Professor Matthew Higgins

**This file contains all reviewer reports in order by version, followed by all author rebuttals in order by version.**

Version 0:

Reviewer comments:

Reviewer #3

(Remarks to the Author)
Summary

The manuscript by Farrell, Cooper, et al., characterizes human antibody responses to the malaria invasion protein PfRIPR and investigates the structural basis for antibody-mediated inhibition of erythrocyte invasion. Using B cells isolated from malaria-exposed individuals in Mali, the authors present a small panel of PfRIPR-binding human monoclonal antibodies that possess high binding affinities but lack growth-inhibitory activity. These three human mAbs are shown to synergize or antagonize the previously identified rodent mAb RP.012. Structural elucidations of each antibody bound to subsections of the invasion complex using X-ray crystallography or cryo-EM, together with biophysical characterization through a FRET-based conformational assay, reveal that the inhibitory antibody RP.012 acts by stabilizing a bent conformation of the PfRIPR EGF6–7 region. In contrast, an antagonist antibody competes with RP.012 binding, while synergistic antibodies bind further along the RIPR tail. The work further expands structural knowledge of PfRIPR within the PfPCRCR complex and demonstrates that antibodies targeting distinct epitopes from RP.012 can exhibit either antagonistic or synergistic effects on parasite growth inhibition. This is a well-executed and well-written study, which provides important insights into rationale design of next-generation malaria blood-stage vaccine candidates.

Major points

The reader would benefit from a better explanation of how the three human mAbs were selected from the cohort of 758 donors. This limited panel size is used to draw conclusions about the functional landscape of PfRIPR-targeting antibodies and while the structural and mechanistic analyses of these antibodies are detailed, the small sample size limits the ability to generalize conclusions about the immunological targeting of PfRIPR, the prevalence of neutralizing versus non-neutralizing epitopes, and the broader implications for vaccine design. At minimum, the authors should clarify what were the selection criteria for these three antibodies, as only three antibodies are actually shown in Ext Fig1 so this sentence does not provide sufficient information regarding the selection process (lines 83-84): “From this small pilot panel of human antibodies, we selected three IgG1 antibodies for further analysis: MAD8-2, MAD8-66 and MAD8-739.”

One important experiment that would substantially strengthen the conclusions of this manuscript would be to apply the FRET-based conformational assay to a broader sampling of human mAbs from the 758 donors. Given the focus on murine RP.012, it would be important to understand if this mechanism applies to human antibodies, which would be relevant to any structure-based antigen engineering the authors propose in the Discussion.

Overall, the cryoEM data processing pipeline looks complex but appropriate. The size of the dataset is impressive and highlights the extent to which the authors pushed to obtain this structure. Nonetheless, improvements are warranted. An additional supplemental figure that shows an enlarged map coloured by local resolution would be important to highlight the map regions of high and low confidence, perhaps also helping to further the authors' point on flexible regions of the complex. In that regard, the model could also benefit from further refinement. Specifically, the poor rotamer % for the PfRIPR-PfCyRPA model is currently 3.12%. Most of these rotamer outliers are in regions with poor map quality so increasing the stereochemistry weights instead of modelling into noise is warranted.

The mechanism presented here (i.e. antibody-induced conformational change in PfRIPR disrupts parasite invasion) is generally in agreement with the mechanism reported in the pre-print from Williams, et al. (doi: https://doi.org/10.64898/2025.12.16.693247), which uses a substantially larger panel of human mAbs to reach a similar conclusion. A difference between the two studies is that Williams, et al., demonstrate synergism through combinations of mAbs which lack any functional growth inhibition, while Farrell, Cooper, et al., propose a mechanism which relies on additions of non-potent human mAbs to a rodent mAb that already possesses functional activity. The authors should consider discussing this study and its implications in the Discussion.

Minor points

Error values for the reported binding kinetics by SPR are missing (Extended data tables 1 and 5).

The difference in elution volumes of RP.012 Fab with RCR or PCR compared to the same complexes with the MAD8 Fabs is not clear from the SEC profiles. Since this is suggested as an indication that RP.012 induces a conformational change in PfRIPR, it would be helpful to the reader if the authors showed comparative elution profiles for each Fab-antigen complex on the same plot, as opposed to separate plots.

When comparing the GIA from RP.012 IgG, Fab, and scFv, it would be important to confirm identical binding kinetics for all 3 molecules – particularly comparing the scFv with the Fab.

Figure 1g would be clearer with an explanation addressing the appearance of cleaved PfRIPR in the gel. Figure 2j would benefit from an alternative orientation of the aligned structures to more clearly illustrate the steric clash between MAD8-2 and RP.012.

A justification for how the resolutions of the X-ray crystallography datasets were determined is missing, as all of the I/s(I) values are below 1.5, two of the CC1/2 values are below 0.5, and two of the structures have very high Rpim values for high resolution shells.

A reference should be provided for the sentence (lines 71-73): “This is a highly endemic region for falciparum, with an estimated two infectious bites per person per day at the peak of the malaria season.”

Lines 75-76: claims of priority such as these are typically not allowed based on journal policies. “From this cohort, we isolated the first human anti-PfRIPR mAbs to date.” Another claim of priority to be fixed is on lines 308-309: “Here we provide the first structural insight into how PfRIPR-targeting antibodies function.” Fixing these statements will in no way diminish the impact of the study.

Unless a Reference can be added to support this statement in lines 301-302, I suggest removing this mention which does not relate to the present study: “while an immunogen based on the ‘top half’ of PfRH5 is under development.”

Reviewer #4

(Remarks to the Author)
Farrell et al. investigate the structural and functional basis of antibody recognition of PfRIPR, an essential component of the PfPCRCR complex that mediates erythrocyte invasion by Plasmodium falciparum. The authors have isolated a panel of human monoclonal antibodies from malaria-exposed individuals and combine these with a previously described inhibitory antibody (RP.012). Using a combination of X-ray crystallography, cryo-EM, FRET-based conformational assays, and parasite growth inhibition assays, the authors show that the inhibitory antibody RP.012 binds at the interface between EGF-like domains 6 and 7 of PfRIPR and induces a pronounced bend in the PfRIPR tail. In contrast, non-inhibitory antibodies binding nearby epitopes do not induce this conformational change.

The study further demonstrates that antibodies targeting PfRIPR can interact either antagonizing or synergizing with one another. In particular, a CTD-binding antibody synergizes with the inhibitory antibody to enhance parasite growth inhibition. The authors propose that PfRIPR tail flexibility is required for invasion and that antibodies such as RP.012 inhibit invasion by stabilizing a non-functional conformation of the PfPCRCR complex.
Overall, the manuscript provides new structural insights into PfRIPR function and antibody-mediated inhibition of erythrocyte invasion, with implications for the design of next-generation blood-stage malaria vaccines, which is an extremely important goal.

Major points:
1. The mechanistic conclusions rely on comparison of a single inhibitory antibody (RP.012) with three non-inhibitory antibodies. Given the central claim that hinge bending underlies inhibition, it would be interesting to analyze additional inhibitory antibodies targeting PfRIPR to determine whether this mechanism is general or specific to RP.012.
2. The central model that bending of the PfRIPR tail inhibits invasion is supported primarily by structural and FRET data. However, direct functional evidence linking hinge conformation to invasion efficiency is limited. It could strengthen the study to test whether the EGF6-7 disulphide-locked mutant affects parasite invasion in cellular assays.
3. The composite model of the PfPCRCR complex relies on integration of experimental structures and AlphaFold

predictions. While reasonable, some of the mechanistic interpretations (e.g., how RP.012 forces the complex into a compact conformation) remain speculative and should be clearly framed as such.

Minor points:
- I find the SPR data confusing. If I understand correct from the methods section, the concentrations for the injections have been in the range of ~3 to 100 nM. At these concentrations, there seems to be practically no dissociation (which of course hints towards a very high affinity), so I don't understand how the Kds have been determined, as for using the kinetic model, one would have to see both association and dissociation. From the steady state values, it would not be possible to determine pM Kd values for these concentation series. This should be explained better, and standard deviations of the Kd determinations should be shown.
- The manuscript would benefit from clearer separation between experimentally determined structures and modelled assemblies in figures.
- The discussion occasionally overstates the certainty of the hinge model; wording could be toned down.
- The description of the antibody isolation strategy could be streamlined for clarity.
- The figures are very dense/buay and hard to grasp and could benefit from being divided into several figures if the journal allows. Also the figures/figure legends could be more informative. For example, it should be possible to understand from the figure and its legend the concentrations used in the SPR injections.
- Some of the structural biology programs used lack references (at least Relion and CryoSPARC).

Reviewer #5

(Remarks to the Author)
I co-reviewed this manuscript with one of the reviewers who provided the listed reports. This is part of the Nature Communications initiative to facilitate training in peer review and to provide appropriate recognition for Early Career Researchers who co-review manuscripts.

Reviewer #6

(Remarks to the Author)
Farrell et al. provides an impressive structural analysis of antibodies bound to PfRIPR, which is important for malaria infection. The structures of antibodies bound to their target epitopes were determined by a combination of X-ray crystallography and cryo-EM. Overall, the data appears to be of good quality, supporting the models and conclusions made in the manuscript. Some additional details would be helpful to clarify and improve the manuscript.
1. The authors indicate that the structure of PfRIPR-MAD8-2 was determined in Relion using BLUSH regularization. The use of AI algorithms is fairly new to cryo-EM and have not been extensively tested. Many of these AI algorithms are trained to 'enhance' maps, which will inherently make maps look better, but it is unclear whether this actual leads to better maps that are accurate. The authors should state in the methods what parameters were used for the analysis, including what initial model was used for refinement and what initial resolution was applied. Furthermore, the authors should include a supplementary figure that compares the map from cryoSPARC (without AI help) to the map from Relion (with BLUSH), with fitting of the atomic model in the map density. The authors should show why the map from Relion (with BLUSH) is better.
2. On line 799, it is stated that the improved map of RIPR-MAD8-2 is at 2.8 A resolution, but this does not match Figure S4. According to the figure, the 2.8 A map is the RIPR(core)-CyRPA structure. The authors should double check the methods and clarify.
3. The provided cryo-EM map of RIPR(core)-CyRPA structure appears to be over sharpened, as seen by the abundance of noise blobs in the map. The authors should provide a less sharpened map, which minimizes noise blobs while maximizing connectivity of the protein density.
4. The authors claim that the smaller complex in the cryo-EM data that appears to be RIPR-MAD8-2 matches the RIPR(tail)-MAD8-2 in the RCR-Cy.007-MAD8-2 structure, which was then combined together to generate the final map of RIPR-MAD8-2. The authors should include a supplementary figure showing why they claim that the two separate structures are the same. Additionally, the authors should include models of the different RIPR(tail) domains fit into the density to show why the authors claim that only one of the domains fit unambiguously into the density.
5. The authors should also deposit and provide the full structure of the RCR-Cy.007-MAD8-2 complex to provide context for the locally refined maps.

Version 1:

Reviewer comments:

Reviewer #3

(Remarks to the Author)
The revised manuscript by Farrell, Cooper, et al. has adequately addressed all points raised.

Reviewer #4

(Remarks to the Author)
I have no further comments.

Reviewer #5

(Remarks to the Author)
I co-reviewed this manuscript with one of the reviewers who provided the listed reports. This is part of the Nature Communications initiative to facilitate training in peer review and to provide appropriate recognition for Early Career Researchers who co-review manuscripts.

Reviewer #6

(Remarks to the Author)
The revisions have greatly enhanced the clarity of the structure components of the manuscript. Some additional points of clarification are included below. The following points would help to promote good standards in cryo-EM data sharing and practice, but do not detract from the readiness of the manuscript for publication.

“We are not sure which maps the reviewer is referring to in this comment.”

My previous comment regarding the over sharpened map is referring to the file “cryosparc_P119_J827_004_volume_map_sharp.mrc” provided by the authors. The map appears over-sharpened, which is a common issue with cryoSPARC. I recommend the authors to sharpen the map with a lower B factor prior to depositing the map in EMDB, which will enhance interpretability for others.

“We considered whether to deposit this composite map and structure but decided against it as we are concerned that it might be misleading to a reader as it does not properly represent the flexibility in the sample. Should a reader wish to generate their own composite map, then they will be able to do so with the data which we will deposit.”

To clarify, I am recommending the authors to deposit the cryo-EM map of the full complex, not the composite atomic model of the complex. The cryo-EM map shows flexibility in the blurred and low-resolution regions of the map.

**Open Access** This Peer Review File is licensed under a Creative Commons Attribution 4.0 International License, which permits use, sharing, adaptation, distribution and reproduction in any medium or format, as long as you give appropriate credit to the original author(s) and the source, provide a link to the Creative Commons license, and indicate if changes were made.
In cases where reviewers are anonymous, credit should be given to 'Anonymous Referee' and the source.
The images or other third party material in this Peer Review File are included in the article’s Creative Commons license, unless indicated otherwise in a credit line to the material. If material is not included in the article’s Creative Commons license and your intended use is not permitted by statutory regulation or exceeds the permitted use, you will need to obtain permission directly from the copyright holder.
To view a copy of this license, visit https://creativecommons.org/licenses/by/4.0/

## REVIEWER COMMENTS

Reviewer #3 (Remarks to the Author):

Summary

The manuscript by Farrell, Cooper, et al., characterizes human antibody responses to the malaria invasion protein PfRIPR and investigates the structural basis for antibody-mediated inhibition of erythrocyte invasion. Using B cells isolated from malaria-exposed individuals in Mali, the authors present a small panel of PfRIPR-binding human monoclonal antibodies that possess high binding affinities but lack growth-inhibitory activity. These three human mAbs are shown to synergize or antagonize the previously identified rodent mAb RP.012. Structural elucidations of each antibody bound to subsections of the invasion complex using X-ray crystallography or cryo-EM, together with biophysical characterization through a FRET-based conformational assay, reveal that the inhibitory antibody RP.012 acts by stabilizing a bent conformation of the PfRIPR EGF6–7 region. In contrast, an antagonist antibody competes with RP.012 binding, while synergistic antibodies bind further along the RIPR tail. The work further expands structural knowledge of PfRIPR within the PfPCRCR complex and demonstrates that antibodies targeting distinct epitopes from RP.012 can exhibit either antagonistic or synergistic effects on parasite growth inhibition. This is a well-executed and well-written study, which provides important insights into rationale design of next-generation malaria blood-stage vaccine candidates.

We thank the reviewer for their accurate summary of our work and for their appreciation of our discoveries.

Major points

The reader would benefit from a better explanation of how the three human mAbs were selected from the cohort of 758 donors. This limited panel size is used to draw conclusions about the functional landscape of PfRIPR-targeting antibodies and while the structural and mechanistic analyses of these antibodies are detailed, the small sample size limits the ability to generalize conclusions about the immunological targeting of PfRIPR, the prevalence of neutralizing versus non-neutralizing epitopes, and the broader implications for vaccine design. At minimum, the authors should clarify what were the selection criteria for these three antibodies, as only three antibodies are actually shown in Ext Fig1 so this sentence does not provide sufficient information regarding the selection process (lines 83-84): "From this small pilot panel of human antibodies, we selected three IgG1 antibodies for further analysis: MAD8-2, MAD8-66 and MAD8-739."

We agree with the reviewer that our description of this panel was not sufficiently comprehensive in the initial version of the manuscript and we inadvertently made it look as though we had isolated a large panel of antibodies and cherry-picked the ones for study. We have rewritten this section of the manuscript (particularly lines 82-87) to make our approach clearer.

In brief, we are currently developing and studying a large panel of PfRIPR targeting antibodies, but this is very challenging due to the scarcity of these antibodies in people who have experienced natural malaria infection. This manuscript focused on a small panel of 7 antibodies isolated through a pilot study. These were from 3 antibody lineages, and we selected one from each lineage for study. The more detailed characterisation of these antibodies and their lineages is now shown in a revised Extended Data Figure 1.

We were delighted that the study of these three antibodies, representative of all of those in our pilot panel, together with RP.012, showed us so much about antibodies function against PfRIPR and that this will already be valuable to guide vaccine development. However, we do not claim that this gives a comprehensive view of the PfRIPR antigenic landscape. We added a few words to lines 424-5 to make this clearer.

One important experiment that would substantially strengthen the conclusions of this manuscript would be to apply the FRET-based conformational assay to a broader sampling of human mAbs from the 758 donors. Given the focus on murine RP.012, it would be important to understand if this mechanism applies to human antibodies, which would be relevant to any structure-based antigen engineering the authors propose in the Discussion.

As mentioned above, it is not the case that a large panel of antibodies is ready for such an analysis as this antibody panel is still in development. When it is, the experiment that the reviewer highlights will be an excellent idea. However, the discovery that antibodies which stabilise bent forms of PfRIPR have GIA is still an important discovery as it already allows us to determine whether bent forms of PfRIPR can be developed which can induce these antibodies, whether or not they turn out to be present in naturally infected people in this specific panel. Therefore, while we acknowledge that this proposed experiment is a very good one, we are not yet ready to conduct it and are not of the view that it is required for this study.

Overall, the cryoEM data processing pipeline looks complex but appropriate. The size of the dataset is impressive and highlights the extent to which the authors pushed to obtain this structure. Nonetheless, improvements are warranted. An additional supplemental figure that shows an enlarged map coloured by local resolution would be important to highlight the map regions of high and low confidence, perhaps also helping to further the authors' point on flexible regions of the complex.

We thank the reviewer for noting that our cryo-EM data processing is appropriately done. We have added an additional Extended Data Figure 5 to provide improved local resolution maps of our two structures (Extended Data Figure 5a and b). Additionally, we show (in Extended Data Figure 5a) an overlay of three different classes, revealing conformational flexibility, and we also include a new supplementary movie to also show the flexibility in this structure.

In that regard, the model could also benefit from further refinement. Specifically, the poor rotamer % for the PfRIPR-PfCyRPA model is currently 3.12%. Most of these rotamer outliers are in regions with poor map quality so increasing the stereochemistry weights instead of modelling into noise is warranted.

We agree with the reviewer and have improved the refinement of this structure, using increased stereochemical restraints. This gives a model with ~1% rotamer outliers, which we have deposited as a replacement model.

The mechanism presented here (i.e. antibody-induced conformational change in PfRIPR disrupts parasite invasion) is generally in agreement with the mechanism reported in the pre-print from Williams, et al. (doi: https://doi.org/10.64898/2025.12.16.693247), which uses a substantially larger panel of human mAbs to reach a similar conclusion. A difference between the two studies is that Williams, et al., demonstrate synergism through combinations of mAbs which lack any functional growth inhibition, while Farrell, Cooper, et al., propose a mechanism which relies on additions of non-potent human mAbs to a rodent mAb that already possesses functional activity. The authors should consider discussing this study and its implications in the Discussion.

We also noted the manuscript by Williams et al, which presents analysis of a panel of antibodies induced through immunisation of a Kymouse. In the past week, an additional study from Xiao et al has also described a set of mouse monoclonal antibodies against PfRIPR. While these studies are in their unreviewed pre-print stage at the time of this response, we agree that it is interesting to compare our findings with theirs. Indeed, the findings of Xiao et al are very similar to ours, with antibodies which show growth-inhibitory activity also stabilising a bent conformation of PfRIPR. The conclusions of Williams et al are a little different as they do not identify growth-inhibitory antibodies, but they still present data which suggests that restricting the conformation mobility of the PfRIPR tail is growth-inhibitory. The reviewer notes that Williams et al show that synergy can occur with combinations of mAbs which lack functional growth inhibition alone. We highlight that we also observe these effects with non-inhibitory antibody pairs, albeit more weakly than for RP.012 (Fig. 4a-b). Williams et al also suggest that their antibodies disrupt the binding of PfRIPR to the PfCSS-PfPTRAMP complex, leading to synergism, which we do not observe. We have added a paragraph to the discussion (lines 387-407) to bring these studies together and to present their shared findings.

Minor points

Error values for the reported binding kinetics by SPR are missing (Extended data tables 1 and 5).

These have now been added.

The difference in elution volumes of RP.012 Fab with RCR or PCR compared to the same complexes with the MAD8 Fabs is not clear from the SEC profiles. Since this is suggested as an indication that RP.012 induces a conformational change in PfRIPR, it would be helpful to the reader if the authors showed comparative elution profiles for each Fab-antigen complex on the same plot, as opposed to separate plots.

We have added an additional panel to Extended Data Figure 2e to show this overlay.

When comparing the GIA from RP.012 IgG, Fab, and scFv, it would be important to confirm identical binding kinetics for all 3 molecules – particularly comparing the scFv with the Fab.

We have added this data to Extended Data Figure 5g and Table 8. We also include additional text in lines 232-3 and 239-41.

Figure 1g would be clearer with an explanation addressing the appearance of cleaved PfRIPR in the gel.

We have added a sentence to clarify that PfRIPR has natural cleavage products (i.e. lines 1360-2).

Figure 2j would benefit from an alternative orientation of the aligned structures to more clearly illustrate the steric clash between MAD8-2 and RP.012.

We have added an additional couple of panels to Extended Data Figure 5f to show a different view of this overlay, as well as a representation of the epitope regions, coloured differently on the surface of PfRIPR.

A justification for how the resolutions of the X-ray crystallography datasets were determined is missing, as all of the I/s(I) values are below 1.5, two of the CC1/2 values are below 0.5, and two of the structures have very high Rpim values for high resolution shells.

In our selection of which cut-off to use for our data, we were guided by studies which show that useful information is found outside the cut-off of CC1/2=0.5 (i.e. https://pmc.ncbi.nlm.nih.gov/articles/PMC4684713/) and have used a cut-off which generates high-quality maps.

A reference should be provided for the sentence (lines 71-73): "This is a highly endemic region for falciparum, with an estimated two infectious bites per person per day at the peak of the malaria season."

We thank the Reviewer for bringing this to our attention. This sentence was based on older data, and we have modified the sentence to reflect a more recent published study that was conducted with the Kalifabougou cohort to calculate infection rates: "This is a highly endemic region for *P. falciparum*, with individuals contracting up to ~50 genetically distinct *P. falciparum* infections over a single 6-month malaria season" and we have added the reference for this finding in lines 74-76.

Lines 75-76: claims of priority such as these are typically not allowed based on journal policies. "From this cohort, we isolated the first human anti-PfRIPR mAbs to date." Another claim of priority to be fixed is on lines 308-309: "Here we provide the first structural insight into how PfRIPR-targeting antibodies function." Fixing these statements will in no way diminish the impact of the study.

We have removed these.

Unless a Reference can be added to support this statement in lines 301-302, I suggest removing this mention which does not relate to the present study: "while an immunogen based on the 'top half' of PfRH5 is under development."

We have added a reference to a review article which proposes the development of RH5top-based immunogens, together with the rationale for their design. We are confident that the statement that 'this is under development' is correct.

Reviewer #4 (Remarks to the Author):

Farrell et al. investigate the structural and functional basis of antibody recognition of PfRIPR, an essential component of the PfPCRCR complex that mediates erythrocyte invasion by Plasmodium falciparum. The authors have isolated a panel of human monoclonal antibodies from malaria-exposed individuals and combine these with a previously described inhibitory antibody (RP.012). Using a combination of X-ray crystallography, cryo-EM, FRET-based conformational assays, and parasite growth inhibition assays, the authors show that the inhibitory antibody RP.012 binds at the interface between EGF-like domains 6 and 7 of PfRIPR and induces a pronounced bend in the PfRIPR tail. In contrast, non-inhibitory antibodies binding nearby epitopes do not induce this conformational change.

The study further demonstrates that antibodies targeting PfRIPR can interact either antagonizing or synergizing with one another. In particular, a CTD-binding antibody synergizes with the inhibitory antibody to enhance parasite growth inhibition. The authors propose that PfRIPR tail flexibility is required for invasion and that antibodies such as RP.012 inhibit invasion by stabilizing a non-functional conformation of the PfPCRCR complex. Overall, the manuscript provides new structural insights into PfRIPR function and antibody-mediated inhibition of erythrocyte invasion, with implications for the design of next-generation blood-stage malaria vaccines, which is an extremely important goal.

We thank the reviewer for the accurate summary of our study and for recognising its importance.

Major points:

1. The mechanistic conclusions rely on comparison of a single inhibitory antibody (RP.012) with three non-inhibitory antibodies. Given the central claim that hinge bending underlies inhibition, it would be interesting to analyze additional inhibitory antibodies targeting PfRIPR to determine whether this mechanism is general or specific to RP.012.

We agree that this is a very interesting question and it is one of our major research objectives. This manuscript describes a first study of a small panel of antibodies, including the first growth-inhibitory monoclonal against PfRIPR to be structurally characterised and the first antibodies derived from humans to be studied.

We are currently undertaking a very large study in which we isolate a substantial panel of antibodies from Malian donors to identify monoclonal antibodies resulting from natural malaria infection which bind to PfRIPR. This is laborious work as growth-inhibitory antibodies against PfRIPR are rare in these individuals and the generation and analysis of this large panel is still therefore underway and will form part of a major future study.

In the meantime, our view is that it is in the interest of the research community to publish this smaller study, as it already provides important novel information to guide PfRIPR vaccine development and design.

2. The central model that bending of the PfRIPR tail inhibits invasion is supported primarily by structural and FRET data. However, direct functional evidence linking hinge conformation to invasion efficiency is limited. It could strengthen the study to test whether the EGF6-7 disulphide-locked mutant affects parasite invasion in cellular assays.

We agree that this is interesting and in previous studies, such as Farrell et al 2024, we have generated such conditional replacement lines to drive mutation of these essential proteins. We did consider doing exactly the experiment that the reviewer suggests but decided against conducting it as part of this study. The main reasons are (i) that PfRIPR has multiple hinge points (see Figure 3d) and so to do this properly we need to understand each of these in structural detail to design a properly controlled study as locking a single hinge in an extended conformation is unlikely to rigidify this region of PfRIPR and (ii) locking the EGF6-7 hinge in a bent conformation to match that found when bound to RP.012 can't be achieved by adding a single disulphide, based on our analysis of the structure. For these reasons, the knowledge in this paper is not sufficient to design a well-considered study of this type and we have decided not to conduct it here. It is something we will return to when we have more insight.

3. The composite model of the PfPCRCR complex relies on integration of experimental structures and AlphaFold predictions. While reasonable, some of the mechanistic interpretations (e.g., how RP.012 forces the complex into a compact conformation) remain speculative and should be clearly framed as such.

We have reviewed our discussion and are confident that this is correctly framed. For example 'is consistent with a model in which' (line 349) or 'may be' (line 350) or 'Alternatively' (line 351) etc.

Minor points:
- I find the SPR data confusing. If I understand correct from the methods section, the concentrations for the injections have been in the range of ~3 to 100 nM. At these concentrations, there seems to be practically no dissociation (which of course hints towards a very high affinity), so I don't understand how the Kds have been determined, as for using the kinetic model, one would have to see both association and dissociation. From the steady state values, it would not be possible to determine pM Kd values for these concentation series. This should be explained better, and standard deviations of the Kd determinations should be shown.

We have improved our presentation of the SPR data, including errors for fitting into the Extended Data tables and better descriptions of the concentration series used and number of replicates into the figure legends. As the errors of fitting for the association and dissociation rates are appropriate, we are confident that we are not over-interpreting these data, which are shown to simply make the point that the antibodies bind tightly. In the one case in which relative binding kinetics are considered, that of the fragments of RP.012, the dissociation rate is more rapid and the fitting is also of high quality, giving us confidence that our analysis is correct.

- The manuscript would benefit from clearer separation between experimentally determined structures and modelled assemblies in figures.

We reviewed our figures and figure legends. While we acknowledge that papers which contain a mixture of experimental structures, AlphaFold models and composites of the two, can be hard to interpret, we think that we have largely made this clear. We have made a few changes to Figure 3c and its legend to improve this labelling but are of the view that otherwise this is clear in the manuscript. If the reviewer points out specific changes which would be useful for clarity, then we are happy to make additional changes.

- The discussion occasionally overstates the certainty of the hinge model; wording could be toned down.

We have checked our discussion of this model in the discussion and have made a few small adjustments to ensure that it is clear that this is a model (see above).

- The description of the antibody isolation strategy could be streamlined for clarity.

We have simplified and clarified our description of the panel (see response to reviewer 1).

- The figures are very dense/buay and hard to grasp and could benefit from being divided into several figures if the journal allows.

We have considered splitting the large figures into multiple panels, to make them less 'busy'. However, each figure contains the data required to provide a biological conclusion and our view is that having these data together in a single figure makes it easier for the reviewer to see how the data comes together to allow conclusions to be drawn. Therefore, while we acknowledge that the figures contain a lot of information, our view is that this is best approach to present our findings.

Also the figures/figure legends could be more informative. For example, it should be possible to understand from the figure and its legend the concentrations used in the SPR injections.

As described above, we have improved the description of the SPR data.

- Some of the structural biology programs used lack references (at least Relion and CryoSPARC).

We have corrected this oversight.

Reviewer #5 (Remarks to the Author):

I co-reviewed this manuscript with one of the reviewers who provided the listed reports. This is part of the Nature Communications initiative to facilitate training in peer review and to provide appropriate recognition for Early Career Researchers who co-review manuscripts.

We thank the reviewer for taking part in the review process and hope that it is an interesting experience.

Reviewer #6 (Remarks to the Author):

Farrell et al. provides an impressive structural analysis of antibodies bound to PfRIPR, which is important for malaria infection. The structures of antibodies bound to their target epitopes were determined by a combination of X-ray crystallography and cryo-EM. Overall, the data appears to be of good quality, supporting the models and conclusions made in the manuscript. Some additional details would be helpful to clarify and improve the manuscript.

We thank the reviewer for their generous comments about our manuscript.

1. The authors indicate that the structure of PfRIPR-MAD8-2 was determined in Relion using BLUSH regularization. The use of AI algorithms is fairly new to cryo-EM and have not been extensively tested. Many of these AI algorithms are trained to 'enhance' maps, which will inherently make maps look better, but it is unclear whether this actual leads to better maps that are accurate. The authors should state in the methods what parameters were used for the analysis, including what initial model was used for refinement and what initial resolution was applied. Furthermore, the authors should include a supplementary figure that compares the map from cryoSPARC (without AI help) to the map from Relion (with BLUSH), with fitting of the atomic model in the map density. The authors should show why the map from Relion (with BLUSH) is better.

We have added an additional panel (Extended Data Figure 4g) to provide a direct side-by-side comparison of these two maps and highlight in the methods (lines 914-6) that this did not alter the overall interpretation of the fit of the model. We have also added the parameters used in refinement to the methods (lines 906-911).

2. On line 799, it is stated that the improved map of RIPR-MAD8-2 is at 2.8 A resolution, but this does not match Figure S4. According to the figure, the 2.8 A map is the RIPR(core)-CyRPA structure. The authors should double check the methods and clarify.

The reviewer is correct in noting that the RIPR-CyRPA structure is at 2.8A resolution. To clarify this, and other aspects of the cryo-EM processing and interpretation, we have extensively revised and expanded their description in both results and methods section, which we hope will avoid this and any other lack of clarity for readers.

3. The provided cryo-EM map of RIPR(core)-CyRPA structure appears to be over sharpened, as seen by the abundance of noise blobs in the map. The authors should provide a less sharpened map, which minimizes noise blobs while maximizing connectivity of the protein density.

We are not sure which maps the reviewer is referring to in this comment. If they are referring to the maps in Figure 3, then these are not sharpened maps, and 'noise' in these maps reflects conformational and compositional heterogeneity as discussed in the main text and methods. We are confident that we have interpreted the maps appropriately. We have added additional representations of maps into the Extended Data Figures and all maps are deposited. We hope that this provides the reviewer with the information they required. If not, then we are happy to make changes in response to specific comments about specific panels.

4. The authors claim that the smaller complex in the cryo-EM data that appears to be RIPR-MAD8-2 matches the RIPR(tail)-MAD8-2 in the RCR-Cy.007-MAD8-2 structure, which was then combined together to generate the final map of RIPR-MAD8-2. The authors should include a supplementary figure showing why they claim that the two separate structures are the same.

We have added additional panels into Extended Data Figures 4 and 5 which we think show this clearly and have also extensively increased the description of the procedure used in both results section and methods.

Additionally, the authors should include models of the different RIPR(tail) domains fit into the density to show why the authors claim that only one of the domains fit unambiguously into the density.

We have added substantially more explanation into this section of the manuscript, as well as new figure panels (i.e. Extended Data Figure 5c and d) to show how we came to our model for the MAD8-2-RIPR structure. While this is laid out now in the manuscript, in brief the data is as follows:

(i) Our epitope binning data (Figure 1f) shows that the binding site for MAD8-2 is in EGF5-6 and so we only need to consider these two domains.

(ii) AlphaFold3 predictions of MAD8.2 bound to EGF5, 6, or 5-6 confidently predict that MAD8.2 binds to EGF6 (Extended Data Figure 5c and Table 7). All predictions with EGF5 have poor metrics, while those including EGF6 have high metrics.

(iii) The AlphaFold predictions were docked onto the experimental maps and only that with EGF6 fits without model protruding from the map (Extended Data Figure 5d).

(iv) Our data that MAD8-2 competes for binding with RP.012 and MAD8-739, which bind to EGF6 is consistent with this interpretation.

5. The authors should also deposit and provide the full structure of the RCR-Cy.007-MAD8-2 complex to provide context for the locally refined maps.

We considered whether to deposit this composite map and structure but decided against it as we are concerned that it might be misleading to a reader as it does not properly represent the flexibility in the sample. Should a reader wish to generate their own composite map, then they will be able to do so with the data which we will deposit.

Reviewer #6 (Remarks to the Author):

The revisions have greatly enhanced the clarity of the structure components of the manuscript. Some additional points of clarification are included below. The following points would help to promote good standards in cryo-EM data sharing and practice, but do not detract from the readiness of the manuscript for publication.

"We are not sure which maps the reviewer is referring to in this comment."

My previous comment regarding the over sharpened map is referring to the file "cryosparc_P119_J827_004_volume_map_sharp.mrc" provided by the authors. The map appears over-sharpened, which is a common issue with cryoSPARC. I recommend the authors to sharpen the map with a lower B factor prior to depositing the map in EMDB, which will enhance interpretability for others.

We already deposited three maps as part of EMD-56282. These include the unsharpened map (*_map.mrc and maps sharpened using either CryoSPARC (*_sharp.mrc) or DeepEMhancer (*_deepemhancer.mrc). The inclusion of the unsharpened map will allow the reader to see the unsharpened data and to sharpen using any B factor of their choice. Indeed, as the map has variable B factor, whichever B factor is used will lead to differences in sharpening across the map, with some appearing areas 'over-sharpened' and some 'under-sharpened'. The provision of the unsharpened map will allow the users to sharpen as they wish depending on the region of the map of interest. Our view is therefore that we have deposited all of the maps which are required for full usefulness for the reader and full transparency.

"We considered whether to deposit this composite map and structure but decided against it as we are concerned that it might be misleading to a reader as it does not properly represent the flexibility in the sample. Should a reader wish to generate their own composite map, then they will be able to do so with the data which we will deposit."

To clarify, I am recommending the authors to deposit the cryo-EM map of the full complex, not the composite atomic model of the complex. The cryo-EM map shows flexibility in the blurred and low-resolution regions of the map.

We can confirm that we have deposited the cryo-EM map of the full complex. In EMD-56281, we include the map of the full complex. In EMD-56282 we also include the map of the full complex, centred around PfRIPR to allow local refinement. In each case, the map of the full complex is available for users. We have only built a model for PfRIPR-PfCyRPA as this is the region of interest for us from this map.
